# Role of Health Information Technology in the Engagement Process of Older Adults’ Cancer Treatment Decision Making

**DOI:** 10.1093/geroni/igaa057.334

**Published:** 2020-12-16

**Authors:** Maryum Zaidi, Priscilla Gazarian, Heather Mattie, Lisa Kennedy Sheldon, C Ann Gakumo

**Affiliations:** 1 UMASS, Boston, Shrewsbury, Massachusetts, United States; 2 University of Massachusetts Boston, Boston, Massachusetts, United States; 3 Harvard T.H. Chan School of Public Health, Boston, Massachusetts, United States; 4 Oncology Nursing Society, Pittsburgh, Pennsylvania, United States; 5 College of Nursing and Health Sciences, Boston, Massachusetts, United States

## Abstract

This study investigates the role of Health Information Technology (HIT) in the process of patient engagement in treatment decision making in older adults in cancer care. Despite the role of HIT in patient engagement processes and government incentives for HIT development, research regarding HIT is lacking among older adults. The following study is a secondary data analysis of a subset of the Health Information National Trend Survey (HINTS 4, Cycle 3), including individuals 65 years old and above. Chi-square tests, logistic regression, and linear regression models were fit to study several sociodemographic, socioeconomic, and psychosocial variables in this study. The results show that education, poverty status, and self-management domain of the patient activation (which is a precursor of the engagement process) were significantly associated with access to and utilization of HIT. No significant differences between access to and utilization of HIT and the diagnosis of cancer were found. However, fatalistic beliefs about the diagnosis of cancer significantly impacted the use of HIT in all models, including those controlling for cancer diagnosis and access to HIT. Specifically, a one-point increase in cancer fatalism score is associated with a 59% decrease in the utilization of HIT, giving evidence that fatalistic beliefs about cancer can drive engagement behaviors regardless of a diagnosis of cancer. Our study provides vital information for providers and policy researchers to take into account for future implementation and development strategies of HIT in cancer care for older adults.

